# Preoperative awareness of nasal and facial aesthetic deformities among rhinoplasty candidates

**DOI:** 10.55730/1300-0144.6099

**Published:** 2025-09-22

**Authors:** Ağah YENİÇERİ, Nagihan GÜLHAN YAŞAR, Burak HAZIR, Melih ÇAYÖNÜ

**Affiliations:** Department of Otorhinolaryngology, University of Health Science, Ankara Bilkent City Hospital, Ankara, Turkiye

**Keywords:** Rhinoplasty, patient, facial aesthetics, self-perception, awareness

## Abstract

**Background/aim:**

This study primarily aimed to evaluate the preoperative awareness of nasal and facial anatomical deformities among patients scheduled for primary rhinoplasty. The secondary objective was to assess changes in patients’ self-perception and aesthetic expectations regarding surgical outcomes following preoperative consultation with the surgeon.

**Materials and methods:**

In this prospective cross-sectional study, 56 adult patients seeking primary aesthetic rhinoplasty at a tertiary care center were included. Each participant completed a 10-item anatomical evaluation form. An experienced rhinoplasty surgeon independently evaluated the same parameters. Additionally, participants completed a two-item visual analogue scale (VAS) questionnaire both before and after the preoperative consultation.

**Results:**

Patients most frequently reported a nasal hump (91%), nasal deviation (89%), and a drooping nasal tip (77%) as their primary concerns. The surgeon most commonly identified a nasal hump (98%), tip ptosis (61%), and nasal deviation (59%). A significant discrepancy was observed between patient and surgeon evaluations of nasal deviation (p < 0.01), as well as in the recognition of extranasal aesthetic regions, particularly the cheek–midface (p = 0.04) and forehead–glabella (p < 0.01) areas. VAS scores for nasal appearance and expected surgical outcomes showed no significant change after the consultation (p = 0.184, p = 0.243).

**Conclusion:**

Although there was general agreement between patients and the surgeon regarding nasal deformities, patient awareness of adjacent facial regions remained limited. Furthermore, preoperative counseling alone was insufficient to alter patients’ self-perception or aesthetic expectations, highlighting the need for enhanced educational strategies in rhinoplasty planning.

## 1. Introduction

The nose plays a central role in facial aesthetics, contributing significantly to perceived facial symmetry, proportion, and individual identity. Even minor deviations or disproportions can disturb facial harmony, prompting individuals to seek surgical correction. Therefore, rhinoplasty holds not only cosmetic but also functional and psychosocial significance in addressing these concerns [1].

Rhinoplasty is among the most commonly performed facial surgeries, addressing both aesthetic concerns and functional nasal problems [2]. Achieving optimal outcomes requires not only precise anatomical assessment and technical execution but also a comprehensive preoperative evaluation that aligns patient expectations with the surgeon’s objectives [2–4].

Despite the numerous rhinoplasty techniques introduced in recent years and the growing expertise in this field, perceptions of surgical success are largely influenced by subjective patient-reported outcomes, with postoperative satisfaction serving as a key determinant [5]. Several standardized instruments have been developed to assess both cosmetic and functional outcomes from the patient’s perspective [6–9].

Numerous studies have reported that patient satisfaction—and consequently rhinoplasty success—can be achieved through a comprehensive understanding of patient expectations by the surgeon prior to surgery [2,5,10,11]. A full understanding of patient expectations requires insight into patients’ awareness of their nasal anatomy and facial aesthetic ideals. Aesthetic success is more likely when both the surgeon and the patient share a mutual understanding of nasal deformities and surgical goals [11]. Furthermore, psychological factors—particularly body dysmorphic disorder (BDD)—are frequently observed among aesthetic rhinoplasty candidates and may significantly influence self-perception and postoperative satisfaction. Therefore, recognizing these psychological aspects is essential for achieving realistic surgical planning and accurate interpretation of outcomes [12–14].

This study primarily aimed to evaluate patients’ preoperative awareness of anatomical deformities involving nasal structures and adjacent facial subregions. A secondary objective was to determine whether detailed preoperative counseling could influence patients’ perceptions of their nasal appearance and their expectations regarding surgical outcomes.

## 2. Materials and methods

Our study included 56 patients who presented to a tertiary healthcare institution for primary aesthetic rhinoplasty. The study was conducted in accordance with the principles of the Declaration of Helsinki, and ethical approval was obtained from the institution’s Clinical Research Ethics Committee (approval number: E1-23-3648). Written informed consent was obtained from all participants for study participation and, when applicable, for the use and publication of their preoperative and postoperative photographs.

This prospectively designed study included adult Turkish patients aged 18 years or older who applied for primary rhinoplasty. Patients who did not provide informed consent, those with a history of nasal trauma within the past year, and patients undergoing revision rhinoplasty were excluded. The manuscript was prepared in accordance with the Strengthening the Reporting of Observational Studies in Epidemiology (STROBE) guidelines.

### 2.1. Anatomical evaluation form

During the preoperative consultation, 10 prominent anatomical features relevant to aesthetic evaluation were identified—those that patients could recognize at first glance: nasal deviation, nasal skin thickness, nasal radix, nasal hump, nasal tip ptosis, nasal tip projection, nostril shape, chin structure, cheek–midface region, and forehead–glabella region. A total of 10 corresponding questions were designed, each offering two response options: “there is a problem” and “no problem”, indicating the presence or absence of deformity in these 10 anatomical regions.

### 2.2. Visual analogue scale

A two-item visual analogue scale (VAS) was incorporated into the evaluation form. In VAS question 1 (VAS Q1), patients were asked to rate the appearance of their current nose on a scale from 0 to 10 (0 = ugliest, 10 = most beautiful). In VAS question 2 (VAS Q2), patients were asked to rate the expected appearance of their nose after surgery on a scale from 0 to 10 (0 = ugliest, 10 = most beautiful). Subsequently, the surgeon informed the patients about the anatomical characteristics of their face and nose, the surgical procedure, and the possible outcomes. After receiving this information, patients were asked to complete VAS Q1 and VAS Q2 again.

### 2.3. Patient assessment procedure

Patients were asked to complete the anatomical assessment form and the VAS before the preoperative consultation. The anatomical parameters included in the assessment form were explained to the patients in detail before they completed the form. Patients used the mirror in the examination room and their available selfie photographs as visual aids while completing the form. Subsequently, the experienced rhinoplasty surgeon who examined each patient completed the 10-item anatomical evaluation form. Patients were asked to complete the two-item VAS again after the consultation. The demographic data of the patients was retrieved from the hospital information system. The patients’ and surgeon’s responses, as well as both sets of VAS scores, were compared. All collected data were analyzed using appropriate statistical methods and interpreted in the context of the current literature.

### 2.4. Statistical analysis

Data analysis was performed using SPSS software version 26.0 (IBM Corp., Armonk, NY, USA). Categorical variables were presented as numbers (n) and percentages (%), whereas continuous variables were expressed as mean ± standard deviation (SD). The one-sample Kolmogorov–Smirnov test was used to assess the distribution of the study variables. The data were normally distributed. The paired-samples t-test was used to compare normally distributed continuous variables, whereas the Pearson’s chi square test was applied to categorical variables. A value of p < 0.05 was considered statistically significant in all comparisons.

## 3. Results

A total of 56 patients were included in the study. The mean age of the participants was 23.7 ± 5.63 years (range: 18–49 years). Of the patients, 45 (80.4%) were female and 11 (19.6%) were male (Table 1).

Patients were most frequently concerned about nasal deviation (n = 50, 89%), nasal hump (n = 51, 91%), and nasal tip ptosis (n = 43, 77%). The cheek–midface (n = 5, 9%) and forehead–glabella regions (n = 3, 5.4%) were considered the least problematic areas by the patients. According to the surgeon’s evaluation, a nasal hump (n = 55, 98%) was the most frequently identified deformity among patients. This was followed by nasal tip ptosis (n = 34, 61%) and nasal deviation (n = 33, 59%) (Table 2).

When the VAS results were analyzed, the mean score for patients’ current nasal appearance in the first question (VAS Q1) was 3.46 ± 1.9. After detailed information was provided by the surgeon, the mean nasal appearance score increased slightly to 3.71 ± 1.83. No statistically significant difference was observed between patients’ evaluations of their current nasal appearance before and after the surgeon’s counselling (p = 0.184). In the second VAS question (VAS Q2), the mean score for the desired nasal appearance after surgery was 9.14 ± 0.92. After detailed information was provided by the surgeon, the mean postoperative nasal appearance score decreased slightly to 9.03 ± 1.02. No statistically significant change was found in patients’ desired nasal appearance before and after the surgeon’s counselling (p = 0.243) (Table 1).

**Figure 1:**
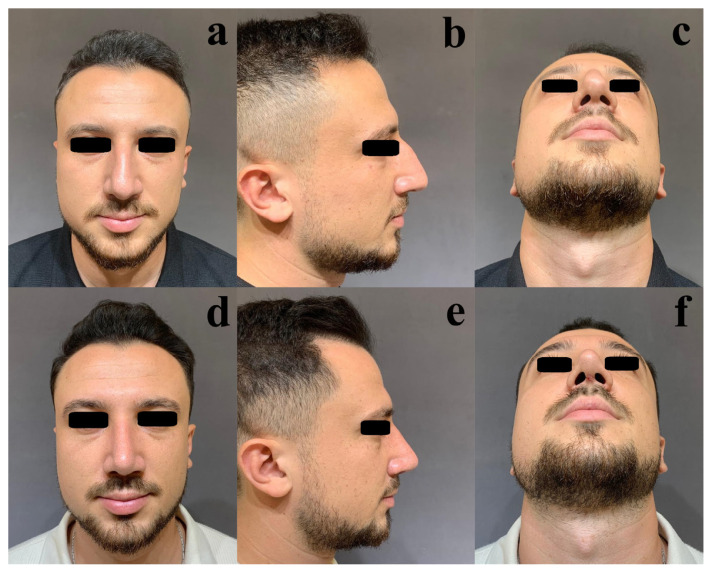
Preoperative (a–c) and postoperative (d–f) frontal, profile, and head-tilt-back views of a 25-year-old male patient who underwent primary rhinoplasty. The right side of the face appears more developed than the left. The configurations of the zygomatic bones also differ.

**Figure 2:**
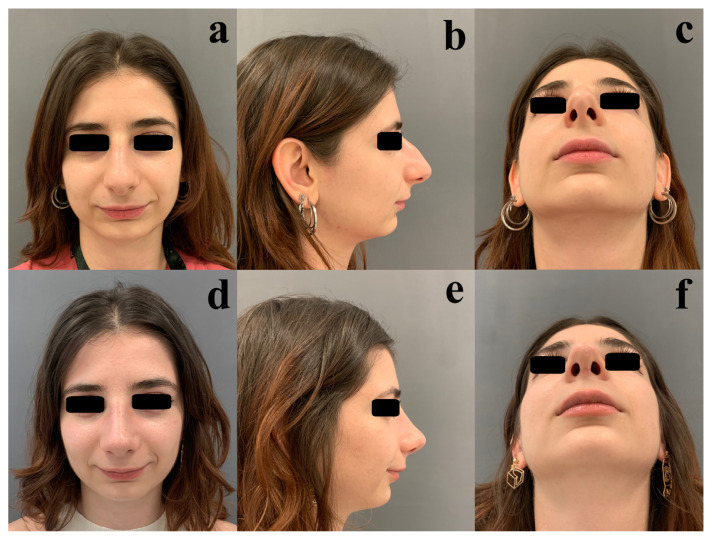
Preoperative (a–c) and postoperative (d–f) frontal, profile, and head-tilt-back views of a 25-year-old female patient who underwent primary rhinoplasty. The patient demonstrates retrognathia.

Statistically significant differences were observed between the patients and the surgeon regarding nasal deviation, as well as the cheek–midface and forehead–glabella regions (p < 0.01, p = 0.04, and p < 0.01, respectively). Examples of aesthetic asymmetries in nonnasal facial regions are presented in Figures 1a–1c and 2a–2c. Postoperative views are shown in Figures 1d–1f and 2d–2f.

Although a difference was identified between the patients and the surgeon regarding nasal tip ptosis, it was not statistically significant (p = 0.067). Agreement between the patients and the surgeon was observed regarding nasal skin thickness, nasal radix, nasal hump, nasal tip projection, nostril shape, and chin structure (Table 2).

## 4. Discussion

The current study revealed three key findings: (1) Unlike the surgeons, most patients believed that they had a nasal deviation; (2) Patients were generally unaware of the anatomical characteristics of facial subregions—specifically the cheek–midface and forehead–glabella regions; and (3) Despite receiving information from the surgeon, there was no statistically significant change in patients’ perceptions of their nasal appearance or their expectations regarding the surgical outcome. These findings highlight the discrepancy between patient perception and clinical reality, emphasizing the need for more effective educational strategies. Moreover, they suggest that conventional preoperative counseling may be insufficient to modify deeply ingrained aesthetic beliefs.

Prerhinoplasty evaluation should be comprehensive, encompassing both functional and aesthetic aspects. Given the variability in patient anatomy and surgical expectations, adopting a standardized approach remains challenging. Nevertheless, a baseline assessment—including nasal airway evaluation, dynamic inspection, and nasofacial analysis from multiple angles—remains essential for optimizing surgical outcomes [4]. This study contributes to this framework by examining patients’ baseline awareness of nasal and facial deformities and by identifying potential gaps between subjective perception and clinical evaluation.

Psychopathological traits are frequently observed among patients seeking aesthetic rhinoplasty, with BDD being particularly prevalent [12]. BDD is characterized by a distorted self-perception and unrealistic aesthetic expectations, often leading to persistent dissatisfaction even after surgery [13,14]. Studies estimate that nearly one-third of rhinoplasty candidates meet the diagnostic criteria for BDD, which can compromise both surgical outcomes and postoperative satisfaction [14]. In light of this risk, preoperative psychological screening is increasingly recommended to identify vulnerable individuals and ensure appropriate patient selection [12]. These psychological and perceptual factors further complicate preoperative aesthetic planning, which must integrate both anatomical analysis and patient insight.

A comprehensive nasofacial analysis—considering age, sex, and ethnic background—is essential for precise aesthetic planning in rhinoplasty [3,4,15]. Optimal outcomes require patients to recognize their nasal deformities, understand surgical limitations, and align their expectations with the surgeon’s clinical judgment. However, this alignment is often hindered by the inherently subjective nature of aesthetic perception, which can create a gap between patients’ perceptions and surgeons’ clinical assessments.

The preoperative consultation serves as a critical stage for aligning the surgeon’s expertise with the patient’s aesthetic expectations [4,16]. Beyond information exchange, its primary goal is to establish a shared understanding of the surgical plan and realistic expectations regarding outcomes. Our findings suggest that this alignment is not always achieved, despite detailed counseling, underscoring the complexity of aesthetic perception among rhinoplasty candidates.

Rhinoplasty candidates often focus narrowly on specific nasal concerns while overlooking the contribution of adjacent facial features to overall facial aesthetics [17,18]. Therefore, surgeons should assess patients’ awareness of facial asymmetries and, when necessary, use tools such as preoperative photographs to clarify surgical objectives and limitations [4]. Enhancing this awareness may improve postoperative satisfaction and reduce the risk of aesthetic, psychological, and social dissatisfaction.

Social media has emerged as a powerful external factor shaping aesthetic ideals and often fosters unrealistic expectations among rhinoplasty candidates [19,20]. Continuous exposure to digitally enhanced images of celebrities and influencers reinforces idealized beauty norms and encourages upward social comparison, which can distort patients’ self-perception and surgical expectations. These dynamics highlight the importance of addressing social media’s influence during preoperative consultations, emphasizing individualized anatomy and realistic outcomes. In this context, our findings underscore the persistence of patient expectations despite counseling, suggesting that external aesthetic influences often outweigh medical explanations.

Discrepancies between patient and surgeon assessments of nasal aesthetics have been well documented [5,10,11,16]. These differences are often attributed to the surgeon’s professional training and refined diagnostic capabilities, which contrast with patients’ more intuitive and global evaluations [5,21]. While patients may accurately recognize major deformities, their ability to identify subtle anatomical variations remains limited. This perceptual gap may contribute to postoperative dissatisfaction, even when surgical outcomes objectively align with clinical goals. Our study quantitatively supports this divergence, particularly regarding the evaluation of nasal deviation and extranasal facial regions.

Interestingly, our findings revealed a notable agreement between patient and surgeon evaluations, particularly regarding nasal structures—an observation that contrasts with previous studies reporting frequent discrepancies. This convergence may reflect a growing societal awareness of nasal aesthetics, potentially shaped by increased exposure to surgical standards and aesthetic ideals through social media. However, nasal deviation remained a major point of disagreement, suggesting that patients may misinterpret structural asymmetries or overestimate their severity. These findings underscore the importance of targeted preoperative counseling to clarify the nature and correctability of nasal deviations.

The aesthetic appearance of the nose can be influenced by the aesthetic characteristics of nonnasal facial structures [17,18]. In our study, inconsistencies were observed between patients and the surgeon in the evaluation of extranasal structures. Statistically significant differences were found between patients and the surgeon in the evaluation of the cheek–midface and forehead–glabella regions. This discrepancy suggests that patients tend to localize their aesthetic concerns to the nose, overlooking the contribution of surrounding facial anatomy to overall nasal aesthetics. These findings highlight a critical gap in patient awareness that may limit their ability to develop realistic expectations, reinforcing the importance of comprehensive facial analysis during consultations.

In our study, patients rated their current nasal appearance significantly lower than their anticipated postoperative outcome. Notably, these scores remained statistically unchanged after detailed preoperative counseling. This result indicates that patients often maintain fixed expectations about their nasal appearance, even after receiving detailed preoperative explanations. Verbal counseling alone appears to be insufficient to modify these expectations, which are likely shaped by personal ideals and social influences. This highlights the challenge of aligning the surgeon’s objective assessment with the patient’s subjective perception and emphasizes the need for more interactive and visual tools to enhance preoperative communication.

Although patients received detailed preoperative counseling, their perceptions of nasal appearance remained largely unchanged. This may be attributed to prior aesthetic experiences, personal ideals of beauty, or social influences that shape and reinforce fixed expectations. Moreover, the inherently subjective nature of aesthetic perception may limit patients’ ability to fully comprehend objective anatomical feedback. Our study did not include visual simulation tools such as image morphing, which might have enhanced patient understanding. Previous studies have demonstrated that image morphing techniques, 3D simulations, and AI-assisted visualization tools can enhance expectation management and help align patients’ perceptions with realistic surgical outcomes [22–25]. In future consultations, personalized morphing examples or before-and-after images of individuals with similar anatomical characteristics could serve as valuable adjuncts to verbal explanations. Furthermore, incorporating brief psychological screening tools into the preoperative evaluation may help identify individuals with body dysmorphic disorder (BDD) or unrealistic expectations and guide the delivery of personalized counseling strategies [12,13].

A key strength of our study was the involvement of a single experienced surgeon in all aesthetic evaluations, which minimized interrater variability and ensured consistency in assessment. However, several limitations should be acknowledged. First, patients were not screened for psychopathological conditions that could have influenced the results of the aesthetic evaluation. In particular, the absence of preoperative psychological screening should be acknowledged as a limitation, especially given this study’s focus on perception and expectation. Second, our study may not be representative of the general population, as it was conducted in a tertiary care center where the majority of participants were female rhinoplasty patients. Third, the selfie photographs that some patients used while completing the anatomical assessment form may have influenced their self-perception. While practical, the use of such photographs may introduce variability due to distortion, lighting conditions, or angle, potentially affecting the accuracy of patients’ self-assessment. Additionally, the visual analog scale (VAS) is inherently subjective; alternative validated patient satisfaction instruments could have been employed.

## 5. Conclusion

Our study revealed that although patients were generally aware of nasal deformities, they lacked awareness of adjacent facial features that influence nasal aesthetics. Despite detailed counseling, patients’ expectations remained unchanged, suggesting that conventional counseling may be insufficient for certain patient groups and that enhanced visual and psychological tools could improve preoperative communication.

## Figures and Tables

**Table 1:** Patient characteristics and visual analogue scale (VAS) results.

Variable	Statistic	p-value
**Patients, N**	56	
**Age (mean ± SD)**	23.7 (±5.63)	
**Sex, N (%)**		
**Female**	45 (80.4%)	
**Male**	11 (19.6%)	
**VAS Q1 (mean ± SD)**		
**Before counselling**	3.46 (±1.9)	0.184
**After counselling**	3.71 (±1.83)	
**VAS Q2**		
**Before information**	9.14 (±0.92)	0.243
**After information**	9.03 (±1.02)	

VAS Q1: Evaluation of current nasal appearance. VAS Q2: Evaluation of expected postoperative nasal appearance. Statistical test: paired samples t-test.

**Table 2:** Anatomical evaluation results.

	Patients		Surgeon		
Anatomical feature	Presence of deformity N (%)	No deformity N (%)	Presence of deformity N (%)	No deformity N (%)	p-value
**Nasal deviation**	50 (89%)	6 (11%)	33 (59%)	23 (41%)	<0.01*
**Nasal skin thickness**	20 (36%)	36 (64%)	18 (32%)	38 (68%)	0.69
**Nasal radix**	24 (43%)	32 (57%)	19 (34%)	37 (66%)	0.33
**Nasal hump**	51 (91%)	5 (9%)	55 (98%)	1 (2%)	0.09
**Nasal tip ptosis**	43 (77%)	13 (23%)	34 (61%)	22 (39%)	0.067
**Nasal tip projection**	31 (55.4%)	25 (44.6%)	27 (48%)	29 (52%)	0.44
**Nostril shape**	26 (46.4%)	30 (53.6%)	20 (36%)	36 (64%)	0.24
**Chin structure**	17 (30.4%)	39 (69.6%)	23 (41%)	33 (59%)	0.23
**Cheek–midface structure**	5 (9%)	51 (91%)	13 (23%)	43 (77%)	0.04*
**Forehead–glabella structure**	3 (5.4%)	53 (94.6%)	31 (55.4%)	25 (44.6%)	<0.01*

Statistical test: Pearson’s chi square test

* p < 0.05.
